# Pneumonectomy in a Child with Multilobar Pneumatocele Secondary to Necrotizing Pneumonia: Case Report and Review of the Literature

**DOI:** 10.1155/2019/2464390

**Published:** 2019-07-17

**Authors:** Christopher A. Gerdung, B. Catherine Ross, Bryan J. Dicken, Candice L. Bjornson

**Affiliations:** ^1^Faculty of Medicine and Dentistry, University of Alberta, Edmonton Clinic Health Academy, 11405-87 Avenue NW, Edmonton, AB, Canada T6G 1C9; ^2^Stollery Children's Hospital, 8440 112 St NW, Edmonton, AB, Canada T6G 2B7; ^3^Cumming School of Medicine, University of Calgary, 3330 Hospital Drive NW, Calgary, AB, Canada T2N 4N1; ^4^Alberta Children's Hospital, 2888 Shaganappi Trail NW, Calgary, AB, Canada T3B 6A8

## Abstract

**Background:**

Community-acquired pneumonia (CAP) is common within pediatrics and contributes disproportionately to morbidity and mortality. Necrotizing pneumonia is a well-documented complication of CAP. It is thought to be caused by necrosis and liquefaction of consolidated lung and can result in damage to lung parenchyma, including pneumatocele development. Management of necrotizing pneumonia with pneumatocele may include hospitalization, intensive care unit admission, and lengthy antibiotic courses. Severe cases may need invasive procedures.

**Case Presentation:**

We present a case of severe necrotizing pneumonia requiring prolonged venovenous extracorporeal membrane oxygenation (V-V ECMO) with development of persistent pneumatoceles, requiring pneumonectomy while on ECMO support to allow for decannulation and extubation.

**Conclusions:**

In critically ill patients with extensive unilateral necrotizing pneumonia with pneumatocele development, surgical intervention can be considered when attempts to wean ventilation have been unsuccessful. This case provides evidence that V-V ECMO and pneumonectomy is a viable salvage therapy in the most critically unwell children.

## 1. Background

Community-acquired pneumonia (CAP) is one of the most common pediatric causes of morbidity and mortality worldwide [1–4]. Necrotizing pneumonia is a severe complication of CAP, thought to be caused by necrosis and liquefaction of consolidated lung, and is often due to *Streptococcus pneumonia* and *Staphylococcus aureus* [5, 6]. Management of necrotizing pneumonia typically includes hospital admission, often with Pediatric Intensive Care Unit (PICU) admission and lengthy antibiotic courses, and may involve invasive procedures including chest tubes, video-assisted thoracoscopic surgery (VATS), or segmental lung resection [6]. Extracorporeal membrane oxygenation (ECMO) is considered a salvage therapy in patients with severe pneumonia, including those with pneumatocele development [7, 8].

## 2. Case Presentation

**Figure 1:**
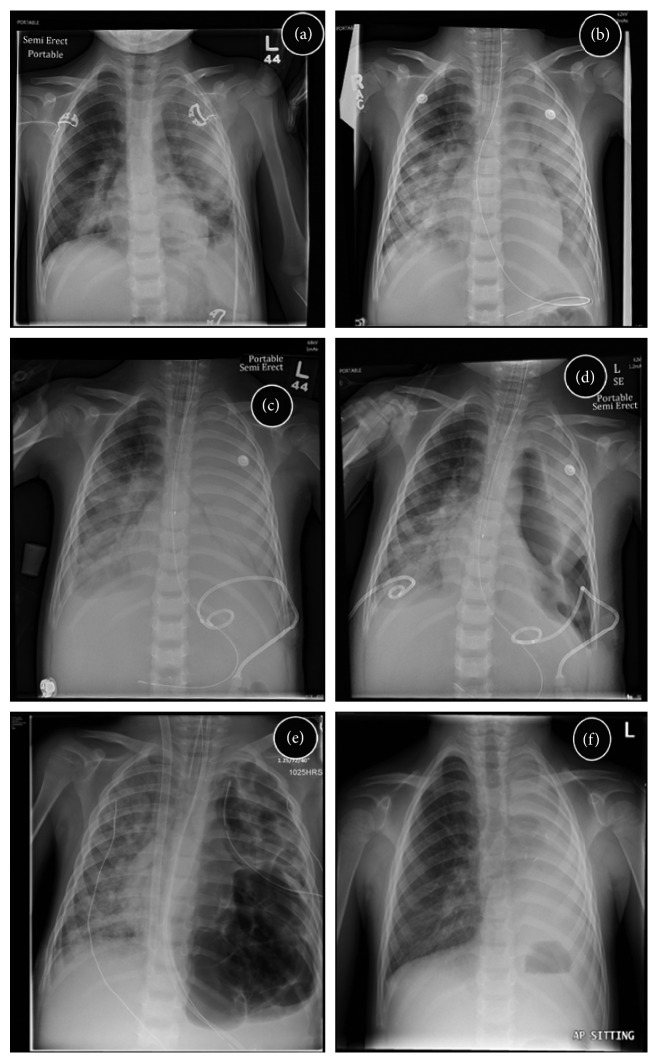
Chest radiographs showing progression of disease. (a) Presentation to ED. (b) Presentation to PICU. (c) Left hemithorax white-out, with percutaneous chest tube. (d) Right pleural effusion following drainage with the percutaneous chest tube. (e) Extensive pneumatocele development prior to pneumonectomy. (f) Resolution of acute disease, following removal of noninvasive respiratory support.

A 4-year-old male presented to the Emergency Department (ED) with a five-day history of dry cough and two-day history of fever, rhinorrhea, and sneezing. Past medical history was significant for premature birth at 35 weeks' gestational age, eczema, food allergies, and one previous hospital admission for wheeze and cough. His presenting vital signs revealed fever (38.9°C), tachypnea (60 breaths/minute), tachycardia (170 beats/min), and oxygen saturations of 70% on room air, with improvement on supplemental oxygen. He had increased work of breathing and intermittent fits of coughing. Chest auscultation revealed decreased breath sounds bilaterally. Chest X-ray showed multifocal pneumonia (Figure 1(a)). Treatment with intravenous ceftriaxone and vancomycin was initiated. Initial investigations revealed leukopenia (2.8 × 10^9^/L), lymphopenia (0.4 × 10^9^/L), anemia (108 g/L), and elevated C-reactive protein (287.2 mg/L). Venous gas was unremarkable other than mild elevation of lactate (lactate 2.7 mmol/L). Nasal secretions were positive for human metapneumovirus.

**Figure 2:**
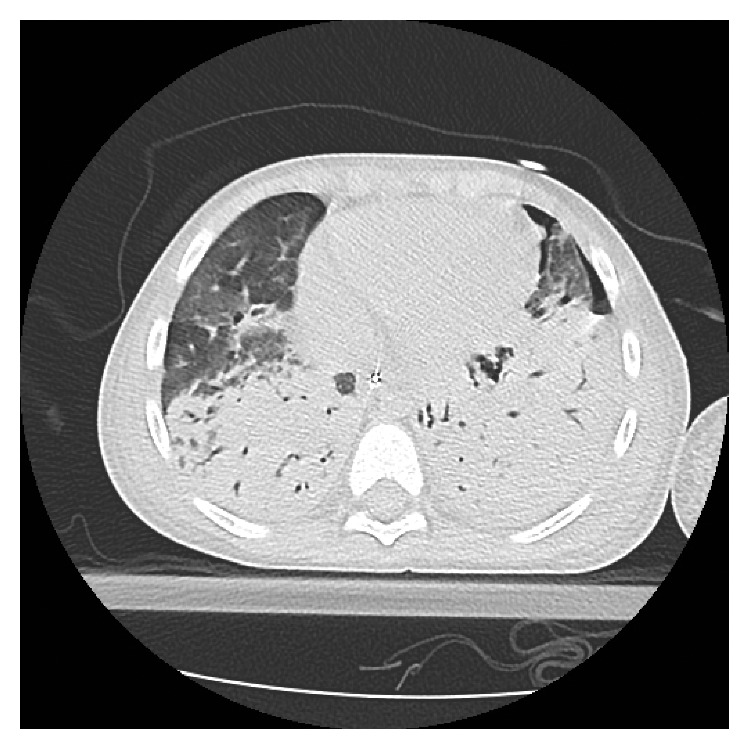
Computed tomography image revealing dense consolidation and pneumothorax. The clinical findings were in keeping with bronchopleural fistula.

Due to worsening work of breathing and tachypnea, the patient was transferred to the PICU on day 2 of admission. He was intubated for type 1 respiratory failure, with worsening bilateral consolidation and development of left pleural effusion (Figure 1(b)). Left chest tube (8.5 French pig-tail catheter) was placed on day 4 of admission following effusion progression (Figure 1(c)), with drainage of cloudy neutrophilic exudate. Blood culture was sterile though 16s rDNA PCR confirmed presence of *Streptococcus pneumoniae* from pleural fluid. Intrapleural fibrinolytics were added, and antibiotic coverage was changed to intravenous ceftriaxone, levofloxacin, and clindamycin. A right pleural effusion developed, and a right chest tube was placed (8.5 French pig-tail catheter). The patient continued to deteriorate, with development of left bronchopleural fistula (Figures 1(d) and 2). High-frequency oscillation (HFO) was initiated; however, he developed mixed respiratory failure with an oxygenation index of 24. Repeat echocardiogram identified pulmonary hypertension (RVSP of 80 mmHg, 2/3 systemic). On day 7 of admission, the patient was initiated on venovenous extracorporeal membrane oxygenation (V-V ECMO) with full heparinization.

While on ECMO, extensive left lung pneumatoceles developed, leaving minimal normal lung parenchyma on the left (Figure 1(e)). Over the subsequent 22 days, attempts to wean ECMO were unsuccessful, as airflow appeared to preferentially ventilate the left lung pneumatoceles rather than the right lung parenchyma capable of gas exchange. This was associated with mixed respiratory failure and need for reinitiation of ECMO. The pneumatoceles increased in size despite ECMO and low ventilation pressures (PS 12 cm H_2_O, PEEP 10 cm H_2_O), resulting in compression of the right lung. Given the persistent compression of viable lung tissue and inability to adequately ventilate, a left pneumonectomy was performed on day 29 of admission while on V-V ECMO. Ex vivo examination revealed large left pneumatoceles, with extensive necrosis and abscess formation. Upon reinitiation of heparin 24 hours after thoracotomy, the patient became hemodynamically unstable and was found to have massive hemothorax postulated to be caused by diffuse oozing throughout the inflamed hemithorax. This necessitated packing of the thoracic cavity, resulting in improved stability. Following pneumonectomy, the patient continued to improve and was successfully decannulated from ECMO on day 33 of admission.

Postpneumonectomy care was complicated by significant critical illness myopathy, as well as a stroke diagnosed clinically on day 39 of admission, presenting with right-sided weakness and left gaze preference during sedation wean. Head MRI revealed left frontal hemorrhage with multiple brain emboli. No arterial thrombus or right to left shunt could be detected on transthoracic echocardiogram to explain the MRI findings; therefore, the stroke was presumed to be secondary to the lengthy ECMO course.

Following ECMO decannulation, ventilator settings were weaned and he was extubated to BiPAP. He developed increased work of breathing within hours of extubation, which was presumed to be due to respiratory muscle weakness secondary to critical illness myopathy, prolonged ventilation, and lengthy courses of sedation and muscle relaxation. There was no evidence to support atelectasis or secondary infection. He was reintubated and continued with sedation wean and intensive rehabilitation. His muscle tone and purposeful movements began to improve. He remained intubated until day 63 of admission, at which point he was successfully extubated to BiPAP, and was discharged on nocturnal BiPAP. Repeat echocardiogram revealed normalization of pulmonary pressures.

Following discharge, he underwent immunologic assessment, with no apparent abnormalities noted within the humoral, cellular, or innate systems. He continues to undergo rehabilitation, and although coordination is slightly reduced on the left, he is able to walk short distances with mobility aids. He continues to make steady functional improvements, and parents have noted no differences in cognition from his baseline. He tolerated increased time off BiPAP during the day and had fully discontinued BiPAP 8 months after presentation. Subsequent polysomnogram was normal. Repeat chest X-ray reveals normalization of the right hemithorax (Figure 1(f)).

## 3. Discussion

We present a case of a 4-year-old child who developed fulminant necrotizing pneumonia necessitating V-V ECMO, who underwent left pneumonectomy due to extensive multilobar pneumatocele expansion that impaired adequate ventilation and ECMO decannulation.

Pneumatoceles are known complications following pneumonia, with a postulated mechanism of pulmonary overinflation secondary to bronchiolar obstruction caused by airway inflammation, poor drainage from necrotic lung parenchyma, and/or collections of air within interstitial tissue [9–12]. Pneumatoceles are commonly observed with *Staphylococcus aureus*; however, other organisms including *Streptococcus*, *H. influenzae*, *Klebsiella*, and *Escherichia coli* have also been implicated [4, 13]. Underlying conditions including anatomic abnormalities and aspiration can predispose patients to recurrent pneumonia and pneumatocele; however, previously healthy individuals can also be affected [14]. The diagnosis is typically made with chest imaging, including chest X-ray and CT, though use of lung ultrasound has also been shown to be effective in the diagnosis of pneumatoceles in the pediatric population [5, 15–17]. Several small studies suggest resolution of simple pneumatoceles by 6 months in most cases although other cases suggest that some pneumatoceles can persist for greater than 12 months without intervention [14, 17, 18]. It is unclear whether coinfection with multiple organisms confers increased risk of developing pneumatocele, or if the clinical presentation of these patients is more severe. Conservative management of pneumatocele is paramount for typically occurring cases and includes use of appropriately dosed antimicrobials to known infectious agents, as well as empiric treatment for *S. pneumoniae* and *S. aureus* [5]. Conservative management alone is unlikely to benefit the patient in situations of cardiorespiratory instability or extensive disease, as death has been reported [14].

Case reports have suggested that treatment can be successful with observation, unilateral ventilation, ventilation with HFO, percutaneous drainage, VATS, lobectomy, and injection of intrapleural fibrin sealant [17, 19–26]. Algorithms have been proposed to aid clinician decision making, which suggest percutaneous needle decompression when the pneumatocele has the following characteristics: (1) occupying greater than 50% of the hemithorax; (2) creating significant atelectasis; (3) bronchopleural fistulae development; (4) tension pneumatocele; or (5) “when follow-up cannot be certain” [17]. This algorithm would likely benefit most patients; however, percutaneous drainage many not benefit all patients. Percutaneous needle decompression is not entirely without risk, as this treatment can lead to the development of bronchopleural fistulae [17]. Additionally, needle decompression may not be successful, thereby necessitating the patient proceed to surgical intervention [17]. Lobectomy is considered a form of definitive management and has been shown to quickly improve the clinical stability of some patients though mortality following this procedure has been reported [24–26].

Pneumonectomy was performed in our patient to obtain adequate source control of the proinflammatory and necrotic lung tissue and to evacuate the large pneumatoceles affecting ventilation. In most situations, removal of as little lung parenchyma as possible is ideal; however, extensive disease may necessitate pneumonectomy to improve cardiorespiratory status. This patient was not suitable for earlier intervention with percutaneous drainage of the pneumatoceles due to the extent of lung disease and high risk of complications associated with full heparinization and ECMO support. Additionally, lobectomy was not suitable, as the amount of salvageable lung tissue was negligible and risked leaving necrotic tissue within the hemithorax. Pediatric pneumonectomy has previously been described in the literature for enlarging pneumatoceles and bronchopulmonary fistula secondary to pneumonia; however, preoperative care was not well described, and treatment with intrapulmonary fibrinolytic agents and HFO ventilation was not performed [27]. Long-term complications following pneumonectomy in children are infrequently reported. Reports suggest most complications are relatively mild and include scoliosis and changes in spirometry values though most children have no marked impairment in exercise or breathing difficulties [28, 29].

This case shows a possible role for V-V ECMO and possible pneumonectomy as a salvage procedure in those patients with extensive disease, who would have previously died.

## 4. Conclusion

In critically ill patients with extensive unilateral disease and with unsuccessful attempts to wean ventilation, further intervention is needed. This case provides evidence that V-V ECMO and pneumonectomy is a viable salvage therapy in the most critically unwell children.

## Abbreviations

BiPAP: Bilevel positive airway pressure
CAP: Community-acquired pneumonia
ECMO: Extracorporeal membrane oxygenation
ED: Emergency department
FiO_2_: Fraction of inspired oxygen
HCO_3_^−^: Bicarbonate
HFO: High-frequency oscillation
IPAP: Inspiratory positive airway pressure
PC: Pressure control
pCO_2_: Partial pressure of carbon dioxide
pO_2_: Partial pressure of oxygen
PEEP: Positive end expiratory pressure
PICU: Pediatric intensive care unit
PS: Pressure support
RVSP: Right ventricular systolic pressure
SIMV: Synchronized intermittent mechanical ventilation
VATS: Video-assisted thoracoscopic surgery
V-V ECMO: Venovenous extracorporeal membrane oxygenation.
